# Examining Subcortical Infarcts in the Era of Acute Multimodality CT Imaging

**DOI:** 10.3389/fneur.2016.00220

**Published:** 2016-12-05

**Authors:** Mindy Y. Q. Tan, Shaloo Singhal, Henry Ma, Ronil V. Chandra, Jamie Cheong, Benjamin B. Clissold, John Ly, Velandai Srikanth, Thanh G. Phan

**Affiliations:** ^1^Stroke and Aging Research Group, Department of Medicine, School of Clinical Sciences at Monash Health, Monash University, Melbourne, VIC, Australia; ^2^Stroke Unit, Monash Medical Centre, Monash Health, Melbourne, VIC, Australia; ^3^Monash Imaging, Monash Health, Melbourne, VIC, Australia

**Keywords:** lacunar, perfusion imaging, angiography, MR imaging, occlusion

## Abstract

**Background:**

Lacunar infarct has been characterized as small subcortical infarct. It is postulated to occur from “*in situ* microatheroma or lipohyalinosis” in small vessel or lacunar mechanism. Based on this idea, such infarcts by lacunar mechanism should not be associated with large area of perfusion deficits that extend beyond the subcortical region to the cortical region. By contrast, selected small subcortical infarcts, as defined by MR imaging in the subacute and chronic stage, may initially have large perfusion deficit or related large vessel occlusions. These infarcts with “lacunar” phenotype may also be caused by disease in the parent vessel and may have very different stroke mechanism from small vessel disease. Our aim is to describe differences in imaging characteristics between patients with small subcortical infarction with “lacunar phenotype” from those with lacunar mechanism.

**Materials and methods:**

Patients undergoing acute CT perfusion/angiography (CTP/CTA) within 6 h of symptom onset and follow-up magnetic resonance imaging (MRI) for ischemic stroke were included (2009–2013). Lacunar infarct was defined as a single subcortical infarct ≤20 mm on follow-up MRI. Presence of perfusion deficits, vessel occlusion, and infarct dimensions was compared between lacunar infarcts and other topographical infarct types.

**Results:**

Overall, 182 patients (mean age 66.4 ± 15.3 years, 66% males) were included. Lacunar infarct occurred in 31 (17%) patients. Of these, 12 (39%) patients had a perfusion deficit compared with those with any cortical infarction (120/142, 67%), and the smallest lacunar infarct with a perfusion deficit had a diameter of <5 mm. The majority of patients with lacunar infarction (8/12, 66.7%) had a relevant vessel occlusion. A quarter of lacunar infarcts had a large artery stroke mechanism evident on acute CTP/CTA. Lacunar mechanism was present in 3/8 patients with corona radiata, 5/10 lentiform nucleus, 5/6 posterior limb of internal capsule (PLIC), 3/5 thalamic infarcts, 1/2 miscellaneous locations. There was a trend to significant with regards to finding lacunar mechanism among patients with thalamic and PLIC infarcts versus lentiform nucleus and corona radiata infarcts (*p* = 0.13).

**Conclusion:**

Diverse stroke mechanisms were present among subcortical infarcts in different locations. When available acute CTP/CTA should be combined with subacute imaging of subcortical infarct to separate “lacunar phenotype” from those with lacunar mechanism.

## Introduction

Lacunar infarct accounts for up to 20% of acute ischemic strokes (1). This infarct is identified based on its subcortical locations using MR imaging. Fisher had listed the location by frequencies as lentiform, caudate, thalamus, pons, posterior limb of internal capsule (PLIC), and convolutional white matter (2). Pathologically, they have been postulated to occur from “*in situ* microatheroma or lipohyalinosis” (3) in small cerebral vessels such as penetrating arteries (2) in the territories of the recurrent artery of Heubner (RAH), lenticulostriate (LA), anterior and posterior choroidal, thalamoperforating, thalamogeniculate, or pontine paramedian arteries (4). Some authors have suggested that lacunar mechanism is different from the usual stroke mechanism and these infarcts may need a different approach to therapy and prevention (5). More recently, it has been controversially suggested that lacunar strokes neither warrant investigation for carotid stenosis (6) nor benefit from thrombolysis with recombinant tissue plasminogen activator (rTPA) (7, 8). It is possible that these proposals may have arisen as result of confusion between infarcts with “lacunar” phenotype and those with lacunar mechanism (9). Infarcts with “lacunar” phenotype are similar to those with lacunar mechanism in that they are located in subcortical region on MR imaging but may differ in other aspects such as perfusion imaging and stroke mechanism.

This confusion between lacunar mechanism and “lacunar phenotype” may have confounded earlier work on this subject (10). More recently, authors have described various patterns of perfusion deficits related to these small subcortical infarcts (11–16). These patterns are categorized as mismatch, matched, and inverse mismatch. In “inverse mismatch” pattern, the latter infarct size is larger than the initial small perfusion deficit (11, 13). However, these perfusion studies of subcortical infarcts did not focus on the angiographic findings (11, 13). Current multimodality imaging with CT perfusion (CTP) and CT angiography (CTA) offers a window into visualizing the associated perfusion deficit and state of the vessel in the hyperacute phase of stroke. Such methods may yield insights into the acute pathophysiology of infarcts that appear to conform to a lacunar appearance on magnetic resonance imaging (MRI) (17) or “lacunar” phenotype as distinct from lacunar mechanism. Our aim was to describe differences in imaging characteristics between patients with small subcortical infarction with “lacunar phenotype” and those with lacunar mechanism.

## Materials and Methods

### Study Sample

Patients with ischemic stroke admitted to the stroke unit at the Monash Medical Centre, Melbourne, Australia, between April 2009 and March 2013 were retrospectively selected through a search of the hospital radiology databases and included if acute CTP/CTA (within 6 h of symptom onset) and follow-up MRI were conducted. Putative clinical stroke mechanism was assigned according to the Trial of ORG 10172 in Acute Stroke Treatment (TOAST) criteria (18). Risk factors and other patient characteristics were obtained from medical records. We excluded patients with hemorrhagic stroke, aneurysmal subarachnoid hemorrhage, and recurrent infarction (which would hinder correlation of CTP and follow-up MRI). This study was approved by the Research Directorate of Monash Health.

### Radiological Data Acquisition

#### CTP and CTA

CT perfusion was acquired in the hyperacute phase using either a General Electric 750HD 64-slice CT scanner or a Philips 128-slice CT scanner with standardized institutional stroke protocols. The images were acquired as two separate 40 mm slabs (total 80 mm coverage from the base of skull to the superior vertex) in shuttle mode with 17 images acquired at every 3.5 s with a total duration of 60 s. Iodinated contrast was administered at 5 ml/s (50 ml in total) followed by a saline flush at 5 ml/s (30 ml in total). CTA was acquired with the iodinated contrast infusion of 5 ml/s (70 ml in total) with the field of view coverage from the aortic arch to the vertex.

#### MRI Brain

Magnetic resonance imaging was performed usually within 90 days of infarct onset on a 1.5 or 3-T superconducting imaging system (General Electric Medical Systems, Milwaukee, WI, USA and Siemens Medical Solutions, Malvern, PA, USA) with echo-planar imaging capability. Imaging series were performed according to the standardized institutional stroke protocols that included T1- and T2-weighted imaging, fluid-attenuated inversion recovery (FLAIR), diffusion-weighted imaging (DWI), susceptibility-weighted imaging (SWI), and MR angiogram with a slice thickness of 4 or 5 mm in the axial plane.

### Imaging Analysis

#### CTP and CTA Analysis

CT perfusion and CTA readers were blinded to clinical stroke mechanism and outcome MRI findings. The CTP was analyzed by an expert stroke neurologist (TP) to identify patients with perfusion deficits. Perfusion deficit was defined as a focal alteration of the mean transit time (MTT) map corresponding to a vascular arterial territory. The unthresholed MTT map was used to identify perfusion deficit. The perfusion analysis was performed using Advantage Windows (GE Medical Systems) and Extended Brilliance Workspace (Philips Healthcare, Best, the Netherlands). Perfusion deficit was categorized as matched, inverse mismatch, and mismatch.

The CTA was analyzed by a single expert neurointerventional radiologist (RC) for occlusions in the following vessels: (1) internal common artery (ICA), basilar artery, vertebral artery; (2) A1 (proximal segment of anterior cerebral artery), M1 [proximal segment of middle cerebral artery (MCA)], P1 (proximal segment of posterior cerebral artery); (3) A2–4 (distal segment of anterior cerebral artery), M2–4 (distal segment of MCA), P2–4 (distal segment of posterior cerebral artery).

#### MRI Analysis

**Figure 1:**
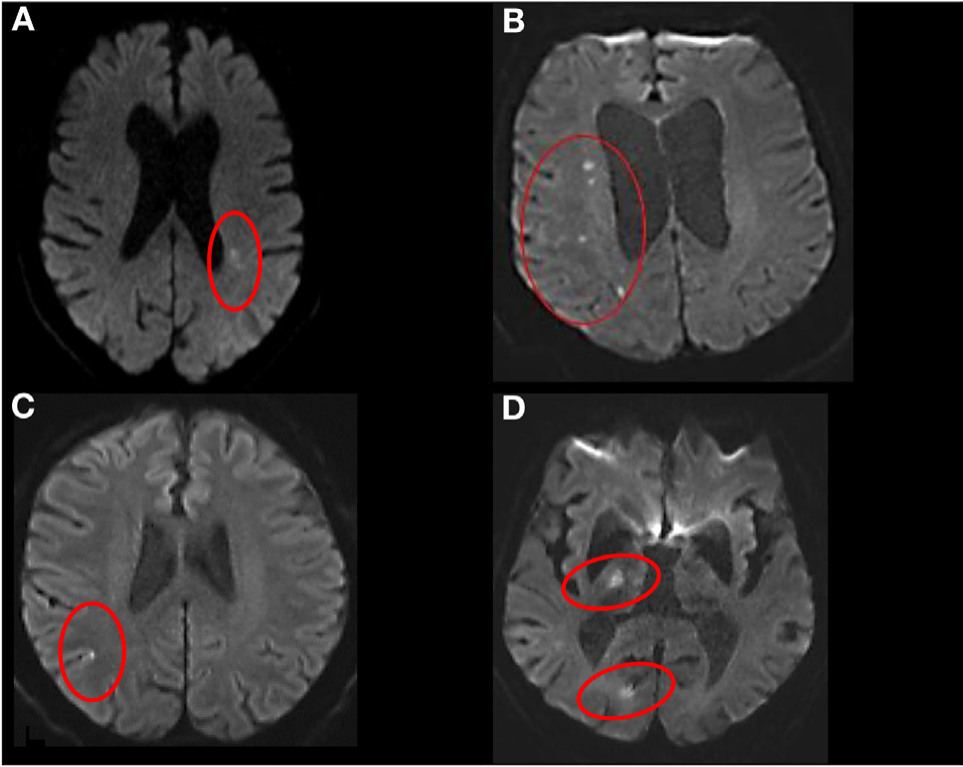
**Stroke topography on diffusion-weighted imaging**. **(A)** Single subcortical, **(B)** multiple subcortical, **(C)** cortical, **(D)** non-confluent cortical–subcortical.

**Figure 2:**
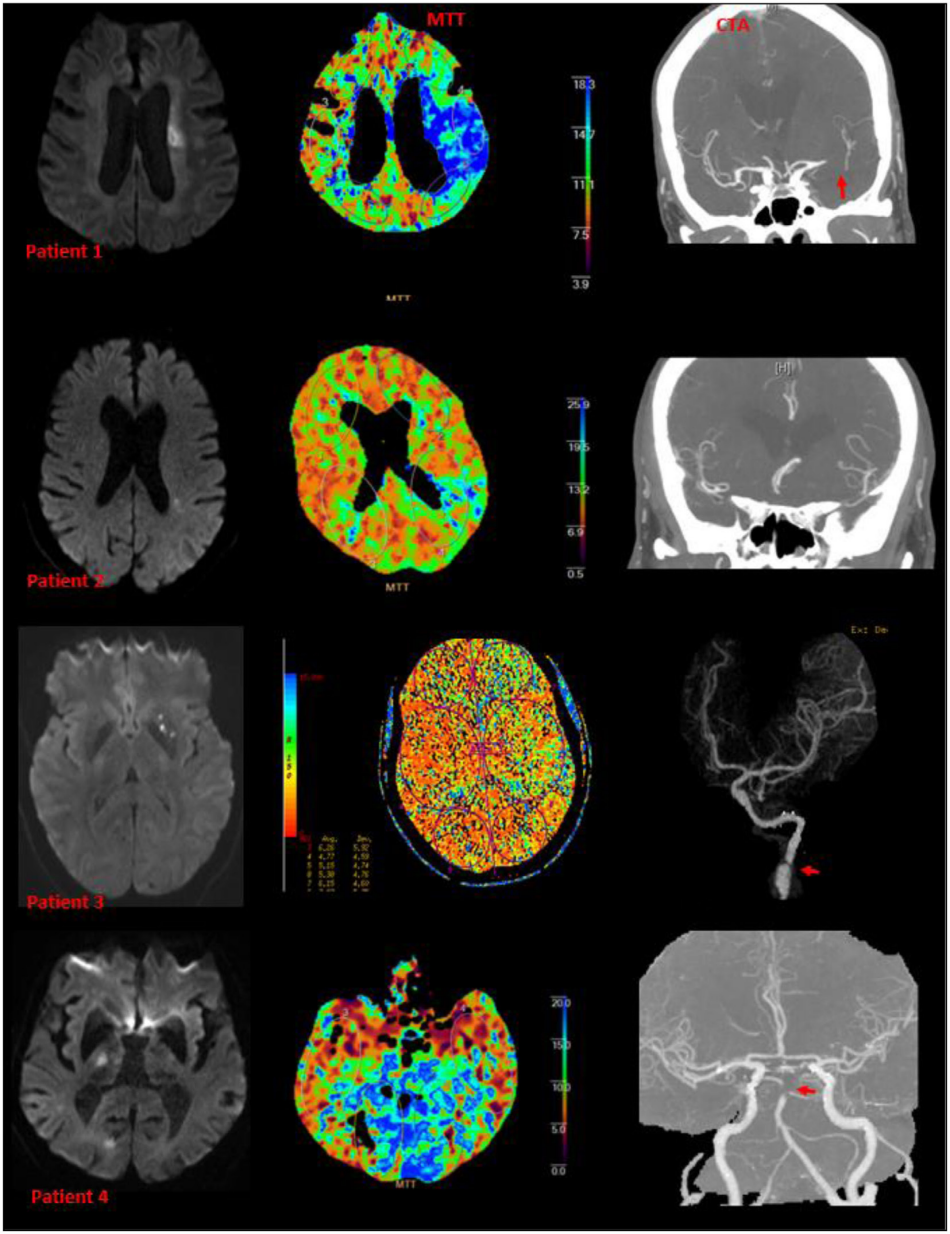
**Examples of small subcortical infarct, perfusion deficit, and large artery diseases**. Patients 1, 3, and 4 have abnormalities on CT angiography (patient 3 had arterial dissection of the internal carotid artery). Patient 2 had M3 branch occlusion on CT angiography.

Magnetic resonance imaging readers were blinded to clinical stroke mechanism and CTP/CTA analysis. The MRI was analyzed by senior stroke research fellow (Shaloo Singhal) who categorized MRI infarcts into five groups: group A, single subcortical infarct; group B, multiple subcortical infarcts; group C, cortical infarcts; group D, non-confluent cortical–subcortical infarcts, and group E, confluent cortical–subcortical infarcts (Figure 1). Subcortical infarct location was assigned if the lesion involved the white or the deep gray matter structures irrespective of dimension. These locations include the brainstem, caudate nucleus, subthalamic nucleus, thalamus, corona radiata, and internal capsule (4). The images of subcortical infarcts were consistent with those used in STandards for ReportIng Vascular changes on nEuroimaging (STRIVE) on subcortical infarcts (Figure 2) (19). Other infarctions, including those located in the cerebellar gray matter, were categorized as cortical. In patients with cortical and subcortical infarcts, the infarcts were termed as either confluent or non-confluent depending on whether they were in close proximity (connected) or distant from each other. A second stroke research fellow (Mindy Y. Q. Tan) measured the maximal diameter of the infarct in the axial plane on DWI.

### Statistical Analysis

The data were analyzed using the R program environment. The chi-squared test for trend in proportions was applied to compare groups for clinical characteristics, infarct dimensions, and presence of relevant perfusion deficits and vessel occlusions. We also assessed the impact of varying infarct dimensions and time to follow-up MRI on the presence of perfusion deficit. A *p*-value of <0.05 was used as the level for statistical significance; Bonferroni corrections were made for multiple comparisons.

## Results

### Patient Characteristics

Between April 2009 and March 2013, 182 patients with ischemic stroke and both acute CTP/CTA and follow-up MRI (mean age 66.4 ± 15.3 years, 66% males) were included. All CTP/CTA was acquired before any reperfusion therapy. The median time from CTP/CTA to follow-up MRI was 15 days (IQR 4.75–35.25). Table 1 details baseline characteristics and TOAST mechanisms. MRI analysis yielded 31 patients with single small subcortical infarcts, 9 with multiple subcortical infarcts, 34 with cortical infarcts, 33 with non-confluent cortical–subcortical infarcts, and 75 with confluent cortical–subcortical infarcts. In group A (single subcortical infarct), 55% of subjects had lacunar mechanism, 26% had large artery mechanism, and 19% had cardioembolic mechanism (Table 1). In group A, infarcts were located in lentiform nucleus 9 (29%), corona radiata 9 (29%), posterior limb of the internal capsule 6 (19%), thalamus 5 (16%), corpus callosum (3%), and brainstem 1 (3%). From group A (single small subcortical infarct) to group E (confluent infarcts), we found a significant increase in proportions with cardioembolic stroke mechanism (*p* for trend = 0.043) and a significant decrease in proportions with undetermined stroke mechanism (*p* for trend < 0.001).

**Table 1:** **Patient characteristics by infarct topography**.

Infarct group		Group A (*n* = 31)	Group B (*n* = 9)	Group C (*n* = 34)	Group D (*n* = 33)	Group E (*n* = 75)	*p* for trend
Demographics	Mean age (years)	64.1 ± 17.1	62.5 ± 21.5	68 ± 12.8	62.9 ± 18.4	68.7 ± 13.1	–
	Male gender	20 (65%)	5 (56%)	28 (82%)	18 (55%)	49 (65%)	0.76
TOAST mechanism	Large vessel	8 (26%)	1 (11%)	5 (15%)	8 (24%)	27 (36%)	0.09
	Cardioembolic	6 (19%)	3 (33%)	15 (44%)	13 (39%)	32 (43%)	0.04
	Lacunar	17 (55%)	0 (0%)	0 (0%)	0 (0%)	0 (0%)	–
	Other	0 (0%)	1 (11%)	1 (3%)	1 (3%)	1 (1%)	0.87
	Undetermined	0 (0%)	4 (44%)	13 (38%)	11 (33%)	15 (20%)	<0.001
Risk factors	Smoker	12 (39%)	4 (44%)	11 (32%)	13 (39%)	27 (36%)	0.82
	Atrial fibrillation	7 (23%)	3 (33%)	15 (44%)	9 (27%)	30 (40%)	0.19
	Ischemic heart disease	6 (19%)	2 (22%)	11 (32%)	9 (27%)	16 (21%)	0.99
	Hypertension	20 (65%)	6 (67%)	22 (65%)	23 (70%)	51 (68%)	0.68
	Hypercholesterolemia	15 (48%)	2 (22%)	18 (53%)	8 (24%)	35 (47%)	0.84
	Diabetes	12 (39%)	1 (11%)	3 (9%)	7 (21%)	21 (28%)	0.72
rTPA-treatment		15 (48%)	6 (67%)	20 (59%)	19 (58%)	38 (51%)	0.89

*Infarct groups: A, single subcortical; B, multiple subcortical; C, cortical only; D, non-confluent cortical–subcortical; E, confluent cortical–subcortical; TOAST, Trial of ORG 10172 in Acute Stroke Treatment; rTPA, recombinant tissue plasminogen activator*.

### CTP Analysis – Perfusion Deficit, Infarct Topography, and Dimension

Perfusion deficits were found in 79% of all patients and in every infarct type (Figure 2; Table 2). Twenty-three patients with subcortical infarcts in groups A and B were imaged on the 64-slice CT scanner. Nine of these 23 patients had perfusion deficit. Seventeen patients in group A were imaged on the 128-slice CT scanner. Nine of these 17 patients had perfusion deficit. There was no statistical difference with regards to perfusion deficit imaged by the different scanners (*p* = 0.4). Twenty-two patients in groups A and B had analysis performed using the delay-sensitive algorithm (11/22 with perfusion deficit), and 18 had analysis performed using the delay-insensitive algorithm (11/18 with perfusion deficit). There was no statistical difference with regards to perfusion deficit analyzed by the different methods (*p* = 0.5).

**Table 2:** **Perfusion deficit and arterial occlusion by infarct topography**.

	Group A (*n* = 31)	Group B (*n* = 9)	Group C (*n* = 34)	Group D (*n* = 33)	Group E (*n* = 75)	*p* for trend
Perfusion deficit	12 (39%)	6 (67%)	24 (71%)	24 (73%)	72 (96%)	<0.001
Sites of arterial occlusion						
ICA, basilar, vertebral	3 (9.6%)	0 (0%)	6 (17.6%)	7 (21.2%)	20 (26.7%)	0.02
A1, M1, P1	4 (12.9%)	3 (33.3%)	6 (17.6%)	6 (18.2%)	28 (37.3%)	0.01*
A2–4, M2–4, P2–4	4 (12.9%)	3 (33.3%)	8 (23.5%)	12 (36.4%)	46 (61.3%)	<0.001*
Perfusion deficit + arterial occlusion	8 (26%)	6 (67%)	14 (42%)	20 (61%)	70 (93%)	<0.001

*Infarct groups: A, single subcortical; B, multiple subcortical; C, cortical only; D, non-confluent cortical–subcortical; E, confluent cortical–subcortical; ICA, internal carotid artery; A1, proximal segment of anterior cerebral artery; M1, proximal segment of middle cerebral artery; P1, proximal segment of posterior cerebral artery; A2–4, distal segment of anterior cerebral artery; M2–4, distal segment of middle cerebral artery; P2–4, distal segment of posterior cerebral artery*.

*p-Value remains significant after Bonferroni correction.

Compared with group A (single subcortical infarcts 12/31, 39% with perfusion deficit), there was an increasing proportion of patients with perfusion deficits going from group B (multiple subcortical infarcts, 6/9, 67%) to group E (confluent cortical subcortical infarcts, 72/75, 96%), *p* for trend <0.001. Perfusion deficit was present in 12/31 (39%) of group A (MRI lacunar infarcts), fewer than those with any cortical infarction (120/142, 67%) *p* < 0.001. Perfusion deficits were present even for small infarcts, seen in 1, 3, 6, and 7 patients with single subcortical infarcts with dimension thresholds ≤5, ≤10, ≤15, and ≤20 mm, respectively. Perfusion deficits were present in 2 of 5 patients with pure motor hemiplegia, 5 of 5 patients with motor-sensory syndrome, and none of 3 patients with dysarthria-clumsy hand syndrome. No patients in this study had pure sensory syndrome.

### CTA Analysis – Perfusion Deficit, Infarct Topography, and Vessel Occlusion

Overall, relevant parent vessel occlusions were present in 118/182 (64.8%) overall, and in 118/138 (89.3%) of patients with perfusion deficits (Table 2). There was a significant increasing likelihood (*p* for trend < 0.001) for the presence of vessel occlusion going from group A (single subcortical) to group E (confluent). Among patients in group A (single subcortical infarcts) with a perfusion deficit, 8/12 (66.7%) had ≥1 occluded vessel, and 6 of these patients had occlusions seen in larger more proximal vessels (4 occluded M1 segment of the MCA, 1 ICA occlusion, and 1 occluded V1 segment of the vertebral artery). Intracranial atheromatous disease was present in 17 of 31 (54.8%) patients in group A. In 8 of the 17 (47.1%) subjects, there was bilateral mild atheromatous disease of the intracranial arteries. Only one patient in this group was classified as having severe stenosis of the M1 artery. However, this stenosis was on the side opposite to the side of the occluded artery and subcortical infarction.

Within group A, large artery or cardioembolic stroke mechanisms were present in 5/10 lentiform nucleus, 5/8 patients with corona radiata, 2/5 thalamic infarcts, 1/6 PLIC, 1/2 miscellaneous locations. There was a trend to significant with regards to finding lacunar mechanism among patients with thalamic and PLIC infarcts versus lentiform nucleus and corona radiata infarcts (*p* = 0.13).

## Discussion

In our study, nearly 40% of patients with small single subcortical infarcts on MRI had perfusion deficits when perfusion studies performed on admissions were examined. Infarcts involving the lentiform nucleus and corona radiata were more likely to be associated with perfusion deficit extending to cortical region. Over one-quarter of these patients had combined perfusion deficit and large artery occlusion on vessel imaging. An important inference from this is that at least a quarter of patients with small single subcortical infarcts do not have lacunar mechanism but have “lacunar” phenotypes with stroke mechanisms, such as large artery or cardioembolic mechanisms. Diverse stroke mechanisms were present among subcortical infarcts in different locations. Subcortical infarcts in the lentiform nucleus or corona radiata may represent “lacunar phenotype” when compared to isolated infarction of the thalamus or PLIC, but this finding was not statistically significant. The clue to the presence of these “lacunar” phenotype and lacunar infarct may be present in the combined use of MR and perfusion and angiographic imaging studies. Use of such data can help the clinician to direct search for stroke mechanism.

### Clinical Lacunar Phenotype

Clinical lacunar phenotype, such as pure motor hemiplegia, do not equate well with lacunar mechanism in our study. Other investigators have made similar findings (10). In this study, only 6 of 19 patients selected clinically on the basis of a “lacunar syndrome” were confirmed on MR imaging as having infarct in subcortical location. Others have reported that clinically defined lacunar syndromes are often inaccurate when compared to MR imaging (20, 21). In this study, the presence of pure motor hemiplegia associated with infarcts involving PLIC was associated with lacunar mechanism, whereas infarcts involving lentiform nucleus or corona radiata could have lacunar mechanism or “lacunar phenotype.”

### Infarct Topography and Vascular Anatomy

In addition to problems discussed above regarding the use of clinical phenotype to define lacunar mechanism, there are issues pertaining to the use of infarct volume for this purpose. It has been suggested that lacunar infarct have an axial diameter of 15 mm on conventional MR imaging or 20 mm on diffusion-weighted MR imaging (22, 23). These subcortical infarcts are similar in locations to those described in the STRIVE, and yet, our study provides supporting evidence of the heterogeneity among subcortical infarcts with regard to stroke mechanism (19). These criteria for lacunar infarcts may apply better with infarct in certain locations (thalamus and PLIC) than others (lentiform nucleus and corona radiata). It should be noted that this relationship was not exclusive and patients with corona radiata and lentiform neucleus can have lacunar mechanism, while patients with thalamic and PLIC infarcts can have large artery or cardioembolic stroke mechanisms. As suggested by a reviewer of this article, there is some controversy as to whether infarcts in the centrum semiovale may meet the MR definition of lacunar infarct (24). For example, investigators of the Secondary Prevention of Small Subcortical Strokes (SPS3) trial and others had considered infarcts in this location to have lacunar mechanism (2, 11, 13, 19, 25–27). However, the centrum semiovale is supplied by the medullary artery of Duret. This artery comes off pial branches of the middle cerebral artery (MCA), and consequently, this region has been suggested to be prone to embolism from large artery or atheromatous disease of the branch arteries or perfusion failure (28). Our data show that a sizeable portion of our patients has at the very least mild intracranial disease.

The LA artery is a perforating artery, and it comes off the proximal segment of the MCA (29). Based on micro-angiographic studies, recent authors have suggested that lacunar infarcts could be due to occlusion of smaller branches of penetrating arteries of the basal ganglia (29). These arterial branches supply functional zone within the basal ganglia and may account for specific stroke syndromes (30). The lack of collaterals among these small arteries may account for the discrete small subcortical infarcts (29). Using arterial templates from this study, our group had previously estimated the size of the infarct related from first- to third-order branches of the penetrating arteries such as the lenticulostiate arteries (31). In that study, the presence of lacunar mechanism was higher among those with infarcts of third-order branches (mean axial diameter ~5.3 mm and volume ~0.7 ml) than infarcts of first order branch (mean axial diameter ~21.2 mm and volume ~20.5 ml) (31). The median size of lacunar infarcts in the SPS3 trials ranged from 0.40 to 0.90 ml (25). A possible inference from these works is that the size of the perfusion deficit would remain confine to subcortical region of the artery and would not spread remote from this arterial territory such as to the cortical region (29, 31). However, if there is occlusion proximal to perforating artery rather than distal part of the perforating artery then perfusion deficit may spread to the cortical region.

### Perfusion Pattern

Our finding of a high frequency of perfusion deficits, in small subcortical infarcts is not surprising since the presence of ischemia should be associated with perfusion deficit (11–16). Several patterns of perfusion deficit with subcortical infarcts have been described including “inverse mismatch.” The inverse mismatch pattern has been described with infarcts involving the thalamus, PLIC, lentiform nucleus, and corona radiata (11–13).

In these cases of “inverse mismatch,” the maximal size attained was ~6 ml and remained located in the subcortical region (11). This infarct volume would approximate to occlusion of second order branches of the penetrating arteries (31).

A key finding of this study on perfusion deficit in subcortical infarct is that the difference between infarcts with “lacunar phenotype” and lacunar mechanism exist in terms of location of infarct and perfusion deficit (2, 25–27). However, the presence of large area of perfusion deficit with infarcts in the lenticular nucleus and corona radiata should with prompt clinicians to evaluate these infarcts carefully. There is agreement among investigators that large striatocapsular infarct may have embolic origin (32). However, there is recent acceptance that small infarcts in this location can have either the lacunar phenotype or have lacunar mechanism (33). Demonstration of perfusion and large vessel disease even in subcortical infarcts ≤5 mm in this study provides support for this notion.

### CTA Findings

The use of CTA has enabled us to define large artery stroke mechanism in 8 of 31 patients with single subcortical infarct (Table 1) and arterial branch occlusion from M2 and beyond in 4 of these subjects (Table 2). This technique is superior to the phase contrast MR angiography (MRA) used in the earlier study (10). Phase contrast MRA can only assess the proximal part of the circle of Willis. Consequently, in that study, it was only able to provide an estimate of occlusive disease of the MCA up to the M2 segment, and hence, it is unsurprising that angiographic abnormalities were not found (10). Other investigators have mentioned conducting angiographic studies (14) or the use of multimodality MR imaging (12, 13) or CT imaging (15), but data on vessel occlusion were not presented.

### Limitations

There are several limitations in this study such as the retrospective nature of the study and the inclusion of only patients who had both CTA/CTP and follow-up MRI. These inclusion criteria potentially led to bias in patient selection. Patients who died very soon after stroke would have been excluded by virtue of not having a follow-up MRI. Such patients would have been more likely to have large cortical infarcts, and hence, this bias is unlikely to affect our findings regarding perfusion deficits in small subcortical infarcts. Similarly, patients with stroke, occurring during sleep, or those with delayed presentation would not have had a CTP according to our institutional imaging protocol. It is possible that if perfusion imaging was performed beyond the 6-h time window, selected patients with perfusion deficit may be found up to 48 h (34). These patients also are more likely to have cortical rather than single subcortical infarcts, and therefore, extending the time window of perfusion imaging may not necessarily lead to significant number of new subcortical infarcts to be included in group A. Similar to other authors (15), we have preferred to use MTT and not use CBF map to identify subcortical perfusion deficit. A potential limitation of this approach is the overestimation perfusion deficit, particularly with the use of unthresholded map. There is possibility of including cortical regions with “benign” oligemia (35). Patients in this study had MR imaging with median time of 2 weeks after the initial CTA and CTP. We had surmised that the small infarcts and perfusion deficit were related as they existed within the same space. There are concerns that the use of CTA and perfusion imaging can delay door to needle time for thrombolytic therapy. That is not the intention of this article. There are strategies such as the administration of rTPA after CT. At the same time as the rTPA is being given, the patients can undergo the remainder of the imaging to determine eligibility for endovascular clot extraction. This article draws attention to the possibility that if these studies have been performed as part of acute stroke protocol imaging then the clinicians should use all information at their disposal.

In summary, single small subcortical infarcts may occur with perfusion deficits and large vessel occlusion in a modest number of patients in whom appropriate care must be taken to identify a proximal embolic source. The use of CTP/CTA in the hyperacute phase of ischemic stroke can be helpful in separating these patients with “lacunar” phenotype from those with lacunar mechanism.

## Author Contributions

Study concept and design: MT, VS, and TP. Acquisition of clinical data: MT and JC. MRI rating: SS. CTA rating: RC. CT perfusion rating: TP. Analysis and interpretation of data: MT, VS, HM, and TP. Drafting of manuscript: MT, HM, JL, BC, VS, and TP. Critical revision of manuscript for intellectual content: all the authors. Statistical analysis: MT and TP.

## Conflict of Interest Statement

TP is on the Advisory Board of Genzyme on Fabry disease and has received payment for lectures including service on speakers’ bureaus for Bayer, Boehringer Ingelheim, Pfizer, and Genzyme. The other authors declare no conflict of interest.
